# Phenolic Compounds from *Halimodendron halodendron* (Pall.) Voss and Their Antimicrobial and Antioxidant Activities

**DOI:** 10.3390/ijms130911349

**Published:** 2012-09-12

**Authors:** Jihua Wang, Jingfeng Lou, Chao Luo, Ligang Zhou, Mingan Wang, Lan Wang

**Affiliations:** 1 College of Agronomy and Biotechnology, China Agricultural University, Beijing 100193, China; E-Mails: nxwjh8121@sina.com (J.W.); azaazafighting88@163.com (J.L.); luochaobest@163.com (C. L.); 2 College of Science, China Agricultural University, Beijing 100193, China; 3 College of Plant Science, Tarim University, Alar 843300, Xinjiang, China; E-Mail: wang-lan95@163.com

**Keywords:** *Halimondendron halodendron*, phenolic compounds, antimicrobial activity, antioxidant activity

## Abstract

*Halimodendron halodendron* has been used as forage in northwestern China for a long time. Its young leaves and flowers are edible and favored by indigenous people. In this study, eleven phenolic compounds were bioassay-guided and isolated from the aerial parts of *H. halodendron* for the first time. They were identified by means of physicochemical and spectrometric analysis as quercetin (**1**), 3,5,7,8,4′-pentahydroxy-3′-methoxy flavone (**2**), 3-*O*-methylquercetin (**3**), 3,3′-di-*O*-methylquercetin (**4**), 3,3′-di-*O*-methylquercetin-7-*O*-β-d-glucopyranoside (**5**), isorhamentin-3-*O*-β-d-rutinoside (**6**), 8-*O*-methylretusin (**7**), 8-*O*-methylretusin-7-*O*-β-d-glucopyranoside (**8**), salicylic acid (**9**), *p*-hydroxybenzoic acid (ferulic acid) (**10**), and 4-hydroxy-3-methoxy cinnamic acid (**11**). They were sorted as flavonols (**1**–**6**), soflavones (**7** and **8**), and phenolic acids (**9**–**11**). Among the compounds, flanools **1**–**4** revealed a strong antibacterial activity with minimum inhibitory concentration (MIC) values of 50–150 μg/mL, and median inhibitory concentration (IC_50_) values of 26.8–125.1 μg/mL. The two isoflavones (**7** and **8**) showed moderate inhibitory activity on the test bacteria. Three phenolic acids (**9**, **10** and **11**) showed strong antibacterial activity with IC_50_ values of 28.1–149.7 μg/mL. Antifungal activities of the compounds were similar to their antibacterial activities. All these phenolic compounds showed significant antimicrobial activity with a broad spectrum as well as antioxidant activity based on 1,1-diphenyl-2-picrylhydrazyl (DPPH) radical scavenging and β-carotene-linoleic acid bleaching assays. In general, the flavonol aglycones with relatively low polarity exhibited stronger activities than the glycosides. The results suggest the potential of this plant as a source of functional food ingredients and provide support data for its utilization as forage as well.

## 1. Introduction

Antioxidants play important roles in scavenging free radicals and/or chain breaking the oxidation reactions both *in vitro* and *in vivo* to minimize oxidative damages to the cells [1]. However, the application of synthetic antioxidants, such as butylated hydroxytoluene (BHT), butylated hydroxylanisole (BHA), and propyl gallate in foods has led to notable adverse effects in consumer health [2,3]. Microbial contamination is another common problem in the food, cosmetic and pharmaceutical industries and the resistance to existing antimicrobial agents creates the need for new antimicrobial compounds. In recent years, there has been an increasing interest in the exploration of naturally occurring antioxidants and antimicrobials from various sources. The plant kingdom produces a diverse array of natural compounds, many of which have strong antioxidant and antimicrobial activities and have been used as food additives, pharmaceuticals and cosmetic components [4]. Phenolic compounds from plants constitute one of the most important groups of natural products with multi-beneficial bioactivities, such as antioxidant [5,6], anti-carcinogenic [7], anti-microbial [8–10], anti-mutagenic [11], anti-inflammatory [12], anti-allergic [13] and anti-obesity [14] properties.

*Halimodendron halodendron* (Pall.) Voss, a leguminous species mainly distributed in the provinces of Xinjiang, Gansu and Inner Mongolia of northwestern China, has been used as forage with good nutrition in desert areas for a long time [15]. The young leaves and flowers are edible and favored by indigenous people [16]. It is possible that *H. halodendron* contains bioactive components that contribute to good health. To the best of our knowledge, there is little information of its chemical composition and bioactivity except for our previous studies about the preparative separation of the main phenolic compounds by high-speed countercurrent chromatography (HSCCC) [17] and bioactivities of the flower essential oil [18]. Other investigations into this plant included a few phylogenic and cytological studies [19,20]. In this context, this study was to isolate and identify the main phenolic compounds from the aerial parts of this plant and to evaluate their antioxidant and antimicrobial activities in order to provide supporting data for the development and utilization of *H. halodendron*.

## 2. Results and Discussion

### 2.1. Elucidation of the Purified Phenolic Compounds

**Figure 1:**
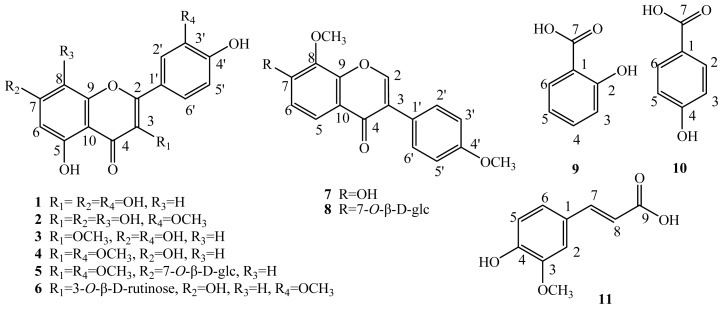
Chemical structures of the compounds (**1**–**11**).

Eleven compounds were bioassay-guided and isolated from the crude ethanol extract of *H. halodendron* (aerial parts) and characterized based on the physicochemical properties and spectral data. Their chemical structures were elucidated based on the spectral data and comparison of the properties and spectral characteristics with those in the literature, which were all known compounds and confirmed as quercetin (**1**) [21], 3,5,7,8,4′-pentahydroxy-3′-methoxy flavone (**2**) [22], 3-*O*-methylquercetin (**3**) [23], 3,3′-di-*O*-methylquercetin (**4**) [24], 3,3′-di-*O*-methylquercetin-7-*O*-β-d-glucopyranoside (**5**) [25], isorhamentin-3-*O*-β-d-rutinoside (**6**) [26], 8-*O*-methylretusin (**7**) [27], 8-*O*-methylretusin-7-*O*-β-d-glucopyranoside (**8**) [28], salicylic acid (**9**) [9], *p*-hydroxybenzoic acid (**10**) [29], and 4-hydroxy-3-methoxy cinnamic acid (ferulic acid) (**11**) [30] (Figure 1). Of the 11 compounds, **1**–**6** were flavonol derivatives, **7** and **8** were isoflavone derivatives, and **9**, **10** and **11** were phenolic acids. Compound **6** should be derived from **4** with the methoxy group at Position 3 being substituted by rutinose, Compound **8** derived from **7** with the hydroxyl group at Position 7 glycosilated with glucose, and Compound **5** derived from **4** with the hydroxyl group at Position 7 glycosilated with glucose.

### 2.2. Antibacterial Activity

**Figure 2:**
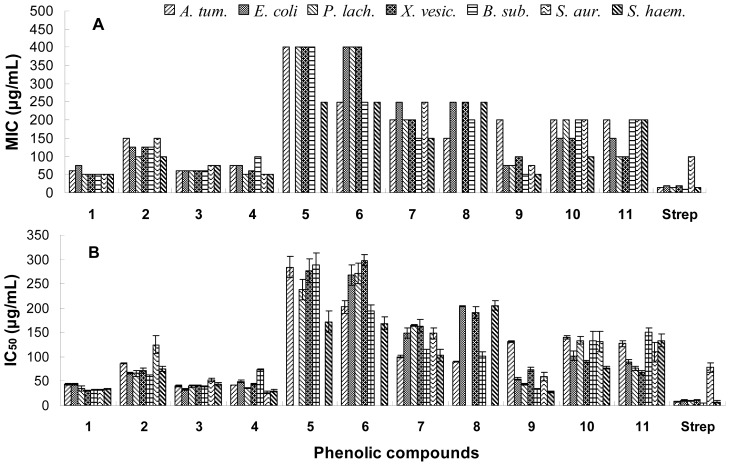
Minimum inhibitory concentration (MIC) (**A**) and median inhibitory concentration (IC_50_) (**B**) of the phenolic compounds from *H. halodendron* on bacteria. *A. tum.: Agrobacterium tumefaciens; E. coli: Escherichia coli; P. lach.: Pseudomonas lachrymans; X. vesic.: Xanthomonas vesicatoria; B. sub.: Bacillus subtilis; S. aur.: Staphyloccocus aureus; S. haem.: Staphylococcus haemolyticus*; Strep: streptomycin sulfate.

All the phenolic compounds were tested for antibacterial activities and their minimum inhibitory concentration (MIC) and median inhibitory concentration (IC_50_) values are reported in Figure 2. Of the six flavonols, only four aglycones, *i.e*. quercetin (**1**), 3,5,7,8,4′-pentahydroxy-3′-methoxy flavone (**2**), 3-*O*-methylquercetin (**3**) and 3,3′-di-*O*-methylquercetin (**4**), revealed strong antibacterial activity with relatively low MIC values of 50–150 μg/mL, and low IC_50_ values of 26.8–125.1 μg/mL. Their glycosides, *i.e*., 3,3′-di-*O*-methylquercetin-7-*O*-β-d-glucopyranoside (**5**) and isorhamentin-3-*O*-β-d-rutinoside (**6**), exhibited weak antibacterial activity with MIC values of 250–400 μg/mL, and IC_50_ values of 168.7–297.7 μg/mL. The two isoflavones 8-*O*-methylretusin (**7**) and 8-*O*-methylretusin-7-*O*-β-d-glucopyranoside (**8**) showed moderate inhibitory activity on the test bacteria. Three phenolic acids (**9**, **10** and **11**) showed strong antibacterial activity with IC_50_ values of 28.1–149.7 μg/mL. There were no obvious differences for the compounds against Gram-positive and negative bacteria.

### 2.3. Antifungal Activity

**Figure 3:**
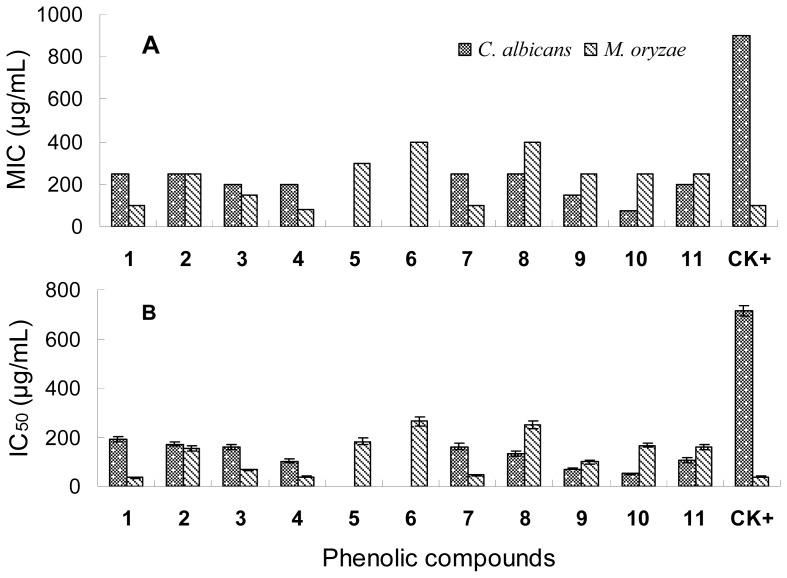
Minimum inhibitory concentration (MIC) (**A**) and median inhibitory concentration (IC_50_) (**B**) of the phenolic compounds from *H. halodendron* on the test fungi. The positive controls (CK+) for *Candida albicans* and *Magnaporthe oryzae* were amphotericin B and carbendazim, respectively.

The antifungal activity of the phenolic compounds with their MIC and IC_50_ values shown in Figure 3 was similar to the antibacterial activity. Compounds **1**, **3**, **4** and **7** had the highest inhibitory activities on *M. oryzae* spore germination with the lowest IC_50_ values of 35.80–67.90 μg/mL. Glycosides **5**, **6** and **8** only exhibited weak activity with relatively high IC_50_ values of 182.9–264.7 μg/mL. The three phenolic acids showed moderate inhibitory activity with IC_50_ values of 99.1–167.5 μg/mL. All tested phenolic compounds exhibited moderate antifungal activity on *Candida albicans*, most of which were even stronger than the positive control amphotericin B, except for Compounds **5** and **6** showing no activity. The results suggest that the fungus *Candida albicans* is an amphotericin-resistant strain and these phenolic compounds are potential antifungal agents against amphotericin-resistant *C. albicans*.

The structure and antimicrobial activity relationships of the six flavonol compounds suggest that aglycones have stronger inhibitory activities on both bacteria and fungi than their glycosides. In addition, Compounds **1**, **3** and **4** also had strong antibacterial activity, suggesting that the hydroxyl or methoxyl groups at the C-3, C-5, C-7, C-3′ and C-4′ positions may be essential to the antibacterial activity of the flavonoids. Indeed, Tsuchiya *et al*. suggested that 2′,4′- or 2′,6′-dihydroxylation of the ring B and 5,7-dihydroxylation of the ring A in the flavanone structure was important for anti-methicillin resistant *Staphylococcus aureus* (MRSA) activity [31]. Kaul *et al*. showed that 4-hydroxy-3-methoxyflavones with a polysubstituted ring A exhibited higher antiviral activity than the same type of compounds with a monosubstituted ring A [32]. Osawa *et al*. also showed, based on the activities detected of several structurally different flavonoids including flavones, flavanones, isoflavones and isoflavanones, that 5-hydroxyflavanones and 5-hydroxyisoflavanones with one, two or three additional hydroxyl groups at the 7, 2′ and 4′ positions could significantly inhibit the growth of *Streptococcus mutans* and *S. sobrinus* [33]. Monosubstitution in ring B was probably contributable to the moderate inhibitory activity of the two isoflavonoids (**7** and **8**) as previously suggested by Kaul *et al*. [32]. Salicylic acid (**9**) showed higher antimicrobial activity than *p*-hydroxybenzoid acid (**10**) except on *Candida albicans*. This suggests that the ortho-substituted hydroxyl group was more important than the para-substituted hydroxyl group for the antimicrobial activity of the phenolic acids.

### 2.4. Antioxidant Activity

**Figure 4:**
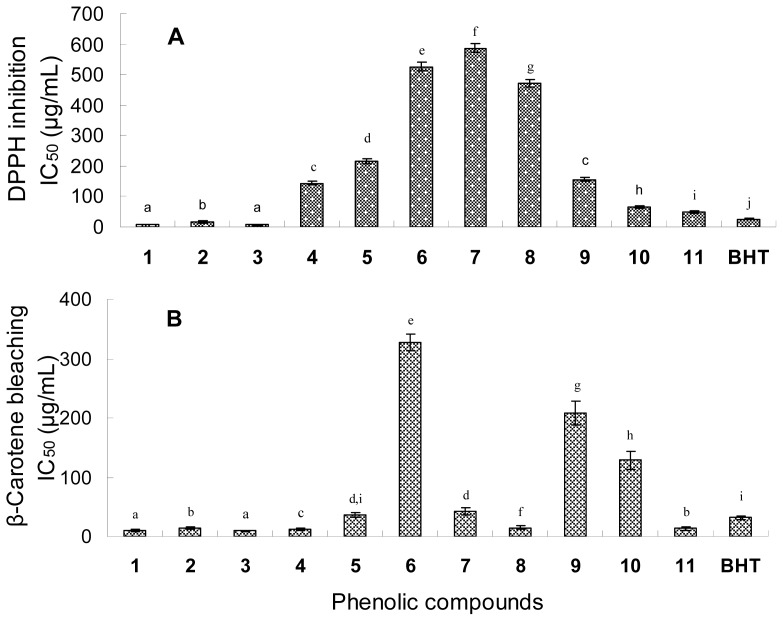
Antioxidant activity of the phenolic compounds of *H. halodendron* measured by 1,1-diphenyl-2-picrylhydrazyl (DPPH) inhibition (**A**) and β-carotene bleaching (**B**) assays. The positive control for the assays was butylated hydroxytoluene (BHT). The error bars represent standard deviations (*n* = 3). Different letters indicate significant differences among the treatments at *p* = 0.05 level.

Figure 4 shows the IC_50_ values of the phenolic compounds in the DPPH radical scavenging and β-carotene-linoleic acid bleaching assays. In view of the IC_50_ values in the DPPH assay, the radical scavenging activities of the six flavonol compounds were in order of quercetin (**1**) (IC_50_ of 7.4 μg/mL) and 3-*O*-methylquercetin (**3**) (IC_50_ of 7.2 μg/mL) > 3,5,7,8,4′-pentahydroxy-3′-methoxy flavone (**2**) (IC_50_ of 16.3 μg/mL) > 3,3′-di-*O*-methylquercetin (**4**) (IC_50_ of 143.9 μg/mL) > 3,3′-di-*O*-methylquercetin-7-*O*-β-d-glucopyranoside (**5**) (IC_50_ of 216.7 μg/mL) > isorhamentin-3-*O*-β-d-rutinoside (**6**) (IC_50_ of 525.5 μg/mL). Three phenolic acids had relatively high radical scavenging activities with relatively low IC_50_ values of 48.0–156.3 μg/mL, while the two isoflavones (**7** and **8**) had very low activities with IC_50_ values of 587.2 μg/mL and 471.5 μg/mL.

In the β-carotene-linoleic acid assay, most of the flavonol compounds exhibited a strong quenching activity with low IC_50_ values of 9.5–36.4 μg/mL except Compound **6** with a high IC_50_ value of 326.5 μg/mL. The three phenolic compounds (**9**, **10** and **11**) also showed relatively high antioxidant activity with IC_50_ of 13.4–128.9 μg/mL. The β-carotene-linoleic acid assay is based on the antioxidant protection of β-carotene from oxidation or bleaching by peroxyl radicals [34]. The antioxidant activity results of the six flavonols from this assay showed a high consistency with those from the DPPH radical assay that aglycones were more favorable than their glycosides. However, the two isoflavones (**7** and **8**) had a strong activity to inhibit the bleaching of β-carotene but low activity in scavenging DPPH radicals.

The structure-antioxidant activity relationships of flavonoids have been widely investigated based on chemical antioxidant activity assays [35–37]. Among the determinants of antioxidant capacity of flavonoids are the presence of a catechol group in Ring B, which is a radical target with stronger electron donating properties, a 2,3-double bond conjugated with the 4-oxo group, which is responsible for electron delocalization, and the presence of hydroxyl groups at C-3 and C-5 positions [38–41]. In our study, Compounds **1** and **3** with the highest antioxidant activities were probably attributable to the 3′,4′-dihydroxy structure, which has a catechol in Ring B. On the other hand, the inhibition potency was not reduced with the substitution of hydroxyl group at C-3 position by methoxy (e.g. compound **3**). Pietta suggested that the presence of a 3-hydroxyl group in Ring C also increased the radical-scavenging activity, while additional hydroxyl or methoxyl groups at C-3, C-5 and C-7 positions of Rings C and A were less important [42]. In other words, the antioxidant activity of natural flavonoids is governed by both the number and the location of their aromatic hydroxyl groups [43]. When those substituted groups were glycosilated, their antiradical activities were obviously lowered, as it was the case for Compounds **5** and **6**, which showed weak radical scavenging activity in this study.

## 3. Experimental Section

### 3.1. Plant Materials

The aerial parts of *Halimodendron halodendron* were collected in June 2008 in Shihezi, Xinjiang, China. The plant was authenticated by Professor Ping Yan at Shihezi University, where the voucher specimen of the plant materials was deposited. The plant materials were left to dry in the shade at room temperature to a constant weight.

### 3.2. Solvents and Chemicals

Beta-carotene, carbendazim, streptomycin sulfate, and 2,2-diphenyl-1-picrylhydrazyl (DPPH) were purchased from Sigma-Aldrich (USA). Linoleic acid was obtained from Johnson Matthey (UK). Amphotericin B and 3-(4,5-dimethylthiazol-2-yl)-2,5-diphenyl tetrazolium bromide (MTT) were purchased from Amresco (USA). Butylated hydroxytoluene (BHT) and Tween-40 were from Beijing Chemical Company. All other unlabelled chemicals and reagents were of analytical grade.

### 3.3. General Analytical Methods

Silica gel (100–200 and 200–300 mesh, Qingdao Marine Chemical Company, China), Sephadex LH-20 (Pharmacia), and C_18_ reversed-phase silica gel (200–300 mesh, YMC) were used for column chromatography (CC). Analytical and preparative thin-layer chromatography (TLC) plates were coated with 0.5 mm and 1 mm layers of silica gel GF_254_, respectively (400 mesh, Qingdao Marine Chemical Company, China). Macroporous resin AB-8 was purchased from Haiguang Chemical Industrial Company (Tianjin, China). The melting point was determined on an XT4-100B microscopic melting-point apparatus (Tianjin Tianguang Optical Instruments Company, China). NMR was recorded on a Bruker-ARX-300 spectrometer (^1^H at 300 MHz and ^13^C at 75 MHz) and Bruker Avance DRX-500 (^1^H at 500 MHz and ^13^C at 125 MHz). Electrospray ionization mass spectrometry (ESI-MS) was performed on a Bruker Esquire 6000 LC/MS spectrometer and electron impact mass spectrometry (EI-MS) on a Thermo Finnigan LCQ mass spectrometer. Absorbance of the samples was measured with a microplate spectrophotometer (PowerWave HT, BioTek Instruments).

### 3.4. Extraction and Fractionation of the Compounds from Plant Materials

The dry aerial parts of *H. halodendron* were ground into powder with a grinder, and 8 kg of the powder was soaked in 95% ethanol at room temperature three times at an interval of 10 days (3 × 30 L). After filtration, the filtrate was concentrated under vacuum at 50 °C, the brown residue (1000 g, yield 12.5%, *w*/*w*) was suspended in water and extracted with petroleum ether and chloroform successively. The chloroform extract (12 g, yield 0.15%, *w*/*w*) was subjected to silica gel column chromatography and eluted with petroleum ether-acetone (from 1:0 to 1:1, *v*/*v*; about 5-fold of the column volume for each eluent), yielding five fractions as detected by TLC. Of these, one fraction (230 mg) was repeatedly chromatographed with silica gel and then purified with Sephadex LH-20 to afford Compounds **4** (16 mg) and **7** (25 mg). The aqueous layer was subjected to macroporous resin AB-8 column chromatography, eluted with water, then with 30% ethanol and finally with 90% ethanol. The volume of each eluent was about five-fold that of the column volume. The fraction eluted out with 90% ethanol was further subjected to silica gel column chromatography eluted with CHCl_3_–MeOH–H_2_O (6:1:0.1, *v*/*v*), yielding five fractions. These fractions were repeatedly chromatographed with silica gel and then purified with Sephadex LH-20 and reverse phase chromatography (RP-18) to afford Compounds **1** (23 mg), **2** (20 mg), **3** (200 mg), **5** (12 mg), **6** (14 mg), **8** (16 mg), **9** (10 mg), **10** (13 mg) and **11** (21 mg).

### 3.5. Physicochemical and Spectrometric Data of the Compounds

Compound **1** was isolated as a yellow amorphous powder (MeOH); mp 314–315 °C; UV (MeOH), λ_max_ 255, 372 nm; ESI-MS *m*/*z* 303 [M + H]^+^, 301 [M − H]^−; 1^H NMR (DMSO-*d*_6_, 300 MHz) δ (ppm): 9.38 (1H, s, OH-3), 12.49 (1H, s, OH-5), 6.19 (1H, d, *J* = 2.0 Hz, H-6), 10.77 (1H, s, OH-7), 6.42 (1H, d, *J* = 2.0 Hz, H-8), 7.68 (1H, d, *J* = 2.2 Hz, H-2′), 9.38 (1H, s, OH-3′), 9.38 (1H, s, OH-4′), 6.90 (1H, d, *J* = 8.5 Hz, H-5′), 7.56 (1H, dd, *J* = 2.2, 8.5 Hz, H-6′); ^13^C NMR (DMSO-*d*_6_, 125 MHz) δ (ppm): 147.9 (C-2), 135.8 (C-3), 176.0 (C-4), 160.8 (C-5), 98.3 (C-6), 164.1 (C-7), 93.5 (C-8), 156.3 (C-9), 103.1 (C-10), 122.1 (C-1′), 115.2 (C-2′), 145.2 (C-3′), 146.9 (C-4′), 115.7 (C-5′), 120.1 (C-6′). Those data were consistent with the literature [21]. Therefore, Compound **1** was identified as quercetin.

Compound **2** was isolated as a yellow amorphous powder (MeOH); mp 272–278 °C; UV (MeOH), λ_max_ 259, 379 nm; ESI-MS *m*/*z* 333 [M + H]^+^, 331 [M − H]^−; 1^H NMR (DMSO-*d*_6_, 300 MHz) δ (ppm): 9.50 (1H, s, OH-3), 12.17 (1H, s, OH-5), 6.27 (1H, *s*, H-6), 9.50 (1H, s, OH-7), 9.50 (1H, s, OH-8), 7.72 (1H, d, *J* = 2.2 Hz, H-2′), 3.84 (3H, s, OCH_3_-3′), 9.50 (1H, s, H-4′), 6.92 (1H, d, *J* = 8.5 Hz, H-5′), 7.61 (1H, dd, *J* = 2.2, 8.5 Hz, H-6′); ^13^C NMR (DMSO-*d*_6_, 125 MHz) δ (ppm): 147.9 (C-2), 135.91 (C-3), 176.1 (C-4), 156.5 (C-5), 98.5 (C-6), 155.5 (C-7), 127.7 (C-8), 148.6 (C-9), 103.1 (C-10), 122.3 (C-1′), 115.1 (C-2′), 145.3 (C-3′), 61.2 (OCH_3_-3′), 146.9 (C-4′), 115.8 (C-5′), 120.1 (C-6′). Those data were consistent with the literature [22]. Therefore, Compound **2** was identified as 3,5,7,8,4′-pentahydroxy-3′-methoxy flavone.

Compound **3** was isolated as a yellow and needle crystal (MeOH); mp 256–257 °C; UV (MeOH), λ_max_ 256, 358 nm; ESI-MS *m/z* 315 [M − H]^−; 1^H NMR (DMSO-*d*_6_, 300 MHz) δ (ppm): 3.78 (3H, s, OCH_3_-3), 12.71 (1H, s, OH-5), 6.20 (1H, d, *J* = 2.0 Hz, H-6), 10.82 (1H, s, OH-7), 6.41 (1H, d, *J* = 2.1 Hz, H-8), 7.56 (1H, d, *J* = 2.2 Hz, H-2′), 10.82 (1H, s, OH-3′), 10.82 (1H, s, OH-4′), 6.92 (1H, d, *J* = 8.5 Hz, H-5′), 7.47 (1H, dd, *J* = 2.3, 8.4 Hz, H-6′); ^13^C NMR (DMSO-*d*_6_, 75 MHz) δ (ppm): 155.8 (C-2), 137.8 (C-3), 178.0 (C-4), 161.4 (C-5), 93.7 (C-6), 164.3 (C-7), 98.7 (C-8), 156.5 (C-9), 104.3 (C-10), 121.0 (C-1′), 115.6 (C-2′), 145.4 (C-3′), 59.8 (OCH_3_-3′), 148.9 (C-4′), 115.9 (C-5′), 120.7 (C-6′). Those data were consistent with the literature [23]. Therefore, Compound **3** was identified as 3-*O*-methylquercetin.

Compound **4** was isolated as a yellow and needle crystal (chloroform); mp 210–212 °C; UV (MeOH), λ_max_ 268, 205 nm; ESI-MS *m*/*z* 329 [M − H]^−^;, 353 [M + Na]^+; 1^H NMR (DMSO-*d*_6_, 300 MHz) δ (ppm): 3.80 (3H, s, OCH_3_-3), 12.68 (1H, s, OH-5), 6.20 (1H, d, *J* = 2.0 Hz, H-6), 10.82 (1H, s, OH-7), 6.48 (1H, d, *J* = 2.1 Hz, H-8), 7.64 (1H, d, *J* = 2.1 Hz, H-2′), 3.86 (1H, s, OCH_3_-3′), 9.88 (1H, s, OH-4′), 6.97 (1H, d, *J* = 8.4 Hz, H-5′), 7.58 (1H, dd, *J* = 2.1, 8.4 Hz, H-6′); ^13^C NMR (DMSO-*d*_6_, 125 MHz) δ (ppm): 155.6 (C-2), 137.9 (C-3), 59.9 (OCH_3_-3), 178.1 (C-4), 161.4 (C-5), 94.0 (C-6), 164.3 (C-7), 98.8 (C-8), 156.5 (C-9), 104.4 (C-10), 122.4 (C-1′), 112.3 (C-2′), 150.0 (C-3′), 55.9 (OCH_3_-3′), 147.6 (C-4′), 115.8 (C-5′), 121.0 (C-6′). Those data were consistent with the literature [24]. Therefore, Compound **4** was identified as 3,3′-di-*O*-methylquercetin.

Compound **5** was isolated as a yellow amorphous powder (MeOH); mp 202–208 °C; UV (MeOH), λ_max_ 255, 355 nm; ESI-MS *m/z* 493 [M + H]^+; 1^H NMR (DMSO-*d_6_*, 500 MHz) δ (ppm): 3.81 (3H, s, OCH_3_-3), 12.58 (1H, s, OH-5), 6.44 (1H, d, *J* = 2.0 Hz, H-6), 6.84 (1H, d, *J* = 2.0 Hz, H-8), 7.65 (1H, d, *J* = 2.0 Hz, H-2′), 3.85 (1H, s, OCH_3_-3′), 6.97 (1H, d, *J* = 8.5 Hz, H-5′), 7.61 (1H, dd, *J* = 2.0, 8.5 Hz, H-6′), 5.05(1H, d, *J* = 7.0 Hz, glc-H-1); ^13^C NMR (DMSO-*d_6_*, 125 MHz) δ (ppm): 156.1(C-2), 138.2 (C-3), 59.9 (OCH_3_-3), 178.3 (C-4), 161.1 (C-5), 99.4 (C-6), 163.1(C-7), 95.0 (C-8), 156.2 (C-9), 106.1 (C-10), 120.8 (C-1′), 112.2 (C-2′), 150.2 (C-3′), 55.9 (OCH_3_-3′), 147.7 (C-4′), 115.8 (C-5′), 122.5 (C-6′), 100.2 (glc-1), 73.3 (glc-2), 77.5 (glc-3), 69.8 (glc-4), 76.7(glc-5), 67.4 (glc-6). Those data were consistent with the literature [25]. Therefore, Compound **5** was identified as 3,3′-di-*O*-methylquercetin-7-*O*-β-d-glucopyranoside

Compound **6** was isolated as a yellow amorphous powder; mp 186–187 °C; UV (MeOH), λ_max_ 355, 255 nm; ESI-MS *m*/*z* 623 [M − H]^−^;, 647 [M + Na]^+; 1^H NMR (DMSO-*d*_6_, 300 MHz) δ (ppm): 12.50 (1H, s, OH-5), 6.19 (1H, d, *J* = 1.9 Hz, H-6), 10.89 (1H, s, OH-7), 6.41 (1H, d, *J* = 1.8 Hz, H-8), 7.86 (1H, d, *J* = 2.0 Hz, H-2′), 3.83 (3H, s, OCH_3_-3′) 9.83 (1H, s, OH-4′), 6.92 (1H, d, *J* = 8.4 Hz, H-5′), 7.53 (1H, dd, *J* = 2.0, 8.4 Hz, H-6′), 5.44 (1H, d, *J* = 7.3 Hz, glc-H-1), 4.41 (1H, d, *J* = 10.8 Hz, rha-H-1), 0.98 (1H, d, *J* =5.6 Hz, rha-H-6); ^13^C NMR (DMSO-*d*_6_, 75 MHz) δ (ppm): 156.7 (C-2), 133.2 (C-3), 177.4 (C-4), 161.4 (C-5), 99.5 (C-6), 165.0 (C-7), 94.3 (C-8), 156.3 (C-9), 103.4 (C-10), 121.2 (C-1′), 113.5 (C-2′), 149.6 (C-3′), 55.9 (OCH_3_-3′), 147.1 (C-4′), 115.5 (C-5′), 122.4 (C-6′), 101.6 (glc-1), 74.5 (glc-2), 76.6 (glc-3), 70.8 (glc-4), 76.1 (glc-5), 67.0 (glc-6), 101.1 (rha-1), 70.5 (rha-2), 70.8 (rha-3), 72.0 (rha-4), 68.5 (rha-5), 17.8 (rha-6). Those data were consistent with the literature [26]. Therefore, Compound **6** was identified as isorhamentin-3-*O*-β-d-rutinoside.

Compound **7** was isolated as a colorless and needle crystal (petroleum ether -acetone); mp 215–216 °C; UV (MeOH), λ_max_ 243, 295 nm; ESI-MS *m*/*z* 321 [M + Na]^+^, 297 [M − H]^−; 1^H NMR (DMSO-*d*_6_, 300 MHz) δ (ppm): 8.43 (1H, s, H-2), 7.74 (1H, d, *J* = 8.8 Hz, H-5), 7.05 (1H, d, *J* = 9.0 Hz, H-6), 10.67 (1H, br s, OH-7), 3.88 (3H, s, OCH_3_-8), 7.53 (1H, d, *J* = 8.8 Hz, H-2′), 7.00 (1H, d, *J* = 8.8 Hz, H-3′), 3.79 (3H, s, OCH_3_-4′), 7.00 (1H, d, *J* = 8.8 Hz, H-5′), 7.53 (1H, d, *J* = 8.8 Hz, H-6′); ^13^C NMR (DMSO-*d*_6_, 75 MHz) δ (ppm): 153.2 (C-2), 123.1 (C-3), 174.8 (C-4), 120.9 (C-5), 115.4 (C-6), 154.9 (C-7), 134.9 (C-8), 60.9 (OCH_3_-8), 150.8 (C-9), 117.6 (C-10), 124.3 (C-1′), 130.2 (C-2′), 113.7 (C-3′), 159.1 (C-4′). 55.3 (OCH_3_-4′), 113.8 (C-5′), 130.2 (C-6′). Those data were consistent with the literature [27]. Therefore, Compound **7** was identified as 8-*O*-methylretusin.

Compound **8** was isolated as a white amorphous powder (MeOH); mp 215–216 °C; UV (MeOH), λ_max_ 204, 255 nm; ESI-MS *m*/*z* 461 [M + H]^+^, 483 [M + Na]^+. 1^H NMR (DMSO-*d*_6_, 300 MHz) δ (ppm): 8.48 (1H, s, H-2), 7.82 (1H, d, *J* = 9.0 Hz, H-5), 7.38 (1H, d, *J* = 9.2 Hz, H-6), 3.94 (3H, s, OCH_3_-8), 7.55 (1H, d, *J* = 9.0 Hz, H-2′), 7.01 (1H, d, *J* = 9.2 Hz, H-3′), 3.82 (3H, s, OCH_3_-4′), 7.01 (1H, d, *J* = 9.0 Hz, H-5′), 7.55 (1H, d, *J* = 9.0 Hz, H-6′), 5.17 (1H, d, *J* = 7.3 Hz, glc-H-1), 4.59 (1H, t, *J* = 6.0 Hz, glc-H-6). ^13^C NMR (DMSO-*d*_6_, 125 MHz) δ (ppm): 154.0 (C-2), 123.0 (C-3), 174.8 (C-4), 120.3 (C-5), 113.9 (C-6), 154.0 (C-7), 136.9(C-8), 60.5 (OCH_3_-8), 149.9 (C-9), 119.3 (C-10), 123.9 (C-1′), 130.0 (C-2′), 113.6 (C-3′), 159.0 (C-4′). 55.1 (OCH_3_-4′), 113.9 (C-5′), 130.0 (C-6′), 100.5 (glc-1), 73.2 (glc-2), 76.7 (glc-3), 69.6 (glc-4), 77.2 (glc-5), 61.2 (glc-6). Those data were consistent with the literature [28]. Therefore, Compound **8** was identified as 8-*O*-methylretusin-7-*O*-β-d-glucopyranoside.

Compound **9** was isolated as a colorless and sheet crystal (lamellar crystal) (acetone); mp 157–158 °C; UV (MeOH) λ_max_ 231, 300 nm; ^1^H NMR (acetone-*d*_6_, 500 MHz) δ (ppm): 6.95 (1H, d, *J* = 9.0 Hz, H-3), 7.54 (1H, m, H-4), 6.93 (1H, t, *J* = 9.0 Hz, H-5), 7.90 (1H, dd, *J* = 9.0, 1.5 Hz, H-6). Those data were consistent with the literature [9]. Therefore, Compound **9** was identified as salicylic acid.

Compound **10** was isolated as a white amorphous powder (MeOH); mp 210–213 °C; UV (MeOH) λ_max_ 204, 252 nm; ^1^H NMR (acetone-*d*_6_, 500 MHz) δ (ppm): 7.92 (2H, d, *J* = 9.0 Hz, H-2 and H-6), 6.92 (2H, d, *J* = 6.92 Hz, H-3 and H-5). Those data were consistent with the literature [29]. Therefore, Compound **10** was identified as *p*-hydroxybenzoic acid.

Compound **11** was isolated as a colorless needle crystal (MeOH); mp 172–174 °C; UV (MeOH), λ_max_ 202, 315 nm; EI-MS *m*/*z* 194 [M]^+; 1^H NMR (DMSO-*d*_6_, 300 MHz) δ (ppm): 7.28 (1H, d, *J* = 2.0 Hz, H-2), 3.84 (3H, s, OCH_3_-3), 6.82 (1H, d, *J* = 8.1 Hz, H-5), 7.09 (1H, dd, *J* = 8.2, 1.9 Hz, H-6), 7.46 (1H, d, *J* = 15.9 Hz, H-7), 6.39 (1H, d, *J* = 15.9 Hz, H-8), 9.55(1H, br s, OH-4), 12.18(1H, br s, COOH). Those data were consistent with the literature [30]. Therefore, Compound **11** was identified as 4-hydroxy-3-methoxy cinnamic acid, also named ferulic acid.

### 3.6. Antibacterial Activity Assay

Antibacterial activity of the compounds was tested against three Gram-positive bacteria (*Bacillus subtilis* ATCC 11562, *Staphylococcus aureus* ATCC 6538 and *Staphylococcus haemolyticus* ATCC 29970) and four Gram-negative bacteria (*Agrobacterium tumefaciens* ATCC 11158, *Escherichia coli* ATCC 29425, *Pseudomonas lachrymans* ATCC 11921 and *Xanthomonas vesicatoria* ATCC 11633). All bacterial species were obtained from the microbial culture stock in the Department of Plant Pathology, China Agricultural University and maintained in LB medium at 28 °C for antibacterial tests. The bacteria were cultured in liquid LB medium (yeast extract 5 g/L, peptone 10 g/L, NaCl 5 g/L, pH 7.0) overnight at 28 °C, and the bacterial suspension was diluted to 1 × 10^6^ cfu/mL for the assay. The antibacterial activity of the compounds was determined with the modified broth dilution-colorimetric assay using the chromogenic reagent 3-(4,5-dimethylthiazol-2-yl)-2,5-diphenyl tetrazolium bromide (MTT) [44]. Briefly, each compound sample was dissolved in ethanol or dimethyl sulfoxide (DMSO) at an initial concentration of 5 mg/mL, and was then diluted with 30% ethanol or 30% DMSO to various concentrations from 0.2 mg/mL to 4.0 mg/mL. The sample solution (10 μL) and the bacterial suspension (90 μL at 1 × 10^6^ cfu/mL) were added into each well of a 96-well microplate. The solvent of sample solution (30% ethanol or 30% DMSO) was included as a negative control and streptomycin sulfate as a positive control in the test. The plates were agitated on a plate shaker and then incubated in the dark at 28 °C for 24 h, at which 10 μL of MTT (5 mg/mL in 0.2 mol/L of pH 7.2 phosphate-buffered saline) was added into each well, and the plates were incubated for another 4 h. The minimum inhibitory concentration (MIC) value was determined as reported previously [45]. For further determination of the IC_50_ values for antibacterial activity of the compounds, the incubated microplate was centrifuged at 1500*g* for 20 min and the supernatant was aspirated. To each well in the microplate, 200 μL of DMSO was added and incubated for 30 min to extract the colored formazan product. After centrifugation of the liquid, the supernatant (100 μL DMSO solution) in each well was transferred to a new 96-well microplate to measure the absorbance at 510 nm on a microplate spectrophotometer. The percentage (%) of bacterial growth inhibition was determined by [(*A*_c_ − *A*_t_)/*A*_c_] × 100, where *A*_c_ and *A*_t_ were the average absorbance of six replicates of the negative controls and that of the compound sample, respectively. The median inhibitory concentration (IC_50_) value was derived from the linear relation between the inhibitory probability and the concentration logarithm [46]. The IC_50_ value was expressed as the mean ± standard deviation of triplicate experiments.

### 3.7. Antifungal Activity Assay

Two fungal species *Candida albicans* and *Magnaporthe oryzae* were selected for antifungal activity assay. The dilution-colorimetric assay was employed to evaluate antifungal activity of the compounds on *Candida albicans* ATCC 10321, which was obtained from the microbial culture stock in the Department of Plant Pathology, China Agricultural University. The *C. albicans* fungus was cultured in liquid potato dextrose (PD) medium overnight at 28 °C, and the fungal suspension was diluted to 1 × 10^6^ cfu/mL. The compound solution was prepared the same as in the above antibacterial activity assay to the final concentrations ranging from 0.05 to 0.30 mg/mL containing 3% ethanol or 3% DMSO. Amphotericin B was used as the positive control and all other procedures were the same as for the antibacterial activity assay.

The spore germination assay was employed to evaluate antifungal activity of the compounds on rice blast fungus, *Magnaporthe oryzae* strain P131 which was provided by Prof. Youliang Peng of the Department of Plant Pathology at China Agricultural University. The fungus was maintained on oatmeal-tomato agar (oatmeal 30 g/L, tomato juice 150 mL/L, and agar 20 g/L) at 25 °C and the fungal spores were prepared from seven-day-old cultures of *M. oryzae* as reported previously [47]. The sample solutions were made to final concentrations ranging from 0.05 to 0.5 mg/mL containing 5% (*v*/*v*) ethanol. The negative control was 5% ethanol and the positive control was carbendazim at concentrations ranging from 0.02 to 0.20 mg/mL. Each treatment was performed in triplicate. The sample or control solution (25 μL) was mixed with an equal volume of spore suspension containing 2 × 10^6^ spores/mL and was then placed on separate concave glass slides. The slides containing the spores were incubated in a moist chamber at 25 °C for 7 h, and then observed under the microscope for spore germination. About 100 spores per replicate were examined for spore germination. The percentage (%) of spore germination inhibition was represented by [(*G*_c_ − *G*_t_)/*G*_c_] × 100, where *G*_c_ and *G*_t_ are the average germinated spore numbers in the negative control and in the sample-treated group, respectively.

### 3.8. Antioxidant Activity Assays

Antioxidant activity of the compounds was determined by DPPH radical scavenging and β-carotene-linoleic acid bleaching assays. DPPH radical scavenging activity was determined based on the reduction of a methanol solution of 1,1-diphenyl-2-picrylhydrazyl (DPPH) as reported by Ono *et al.* [48]. Briefly, 80 μL of DPPH solution (0.2 mg/mL) and 20 μL of each phenolic compound solution in 30% ethanol were added into each well of a 90-well microplate. The microplate was shaken vigorously for complete mixing of the solution and left to stand at 37 °C for 30 min in the dark. The absorbance of the solution was then measured at 515 nm on a microplate spectrophotometer. Percentage (%) of DPPH radical scavenging activity was calculated by [(*A*_control_ − *A*_sample_)/*A*_control_] × 100, where *A*_control_ is the absorbance of the control containing all reagents except the test sample, and *A*_sample_ is the absorbance of the test sample. BHT was used as the positive control. The tests were performed in triplicate.

The β-carotene-linoleic acid bleaching assay of antioxidant activity was performed as reported previously [4]. Briefly, 25 μL linoleic acid and 200 mg Tween-40 were added into a β-carotene solution (0.5 mg β-carotene dissolved in 1 mL chloroform), followed by removal of the chloroform in a rotary evaporator at 50 °C. The remaining mixture was mixed with 50 mL of oxygen-saturated distilled water by shaking vigorously. The β-carotene-linoleic acid-Tween mixture solution (90 μL) and the sample compound solution (10 μL at various concentrations from 0.05 mg/mL to 4.0 mg/mL) were added into each well of a 90-well microplate. An equal volume of 30% ethanol was used as the blank. The microplate was placed in an incubator at 50 °C for 2 h together with BHT as the positive control. The absorbance solution was measured at 460 nm on a microplate spectrophotometer. The β-carotene bleaching inhibition activity of each sample was represented by the ratio of the absorbance of the mixture solution after 2 h of incubation to that of the initial mixture. All tests were performed in triplicate.

### 3.9. Statistical Analysis

The data were collected and expressed as the mean ± standard deviation of three independent experiments. Analysis of variance (ANOVA) and the significance test of treatment effects were determined by Duncan’s multiple range test at *p* = 0.05 level.

## 4. Conclusions

In this study, eleven phenolic compounds including six flavonols (**1**–**6**), two isoflavones (**7** and **8**), and three phenolic acids (**9**–**11**) were bioassay-guided and isolated from the aerial parts of *H. halodendron*, and showed a broad spectrum of antimicrobial activity on several microorganisms including bacteria (Gram-positive and negative) and fungi and as well as notable antioxidant activities based on two chemical assays. They should be the main active compounds in the crude ethanol extract of the aerial parts of this plant. In general, the flavonoid aglycones with relatively low polarity had higher antimicrobial and antioxidant activities than the glycosides with high polarity. The study suggests the potential of the *H. halodendron* plant as a source of functional food ingredients with antimicrobial and antioxidant activities. It also provides new data useful for the utilization and further development of *H. halodendron* aerial parts as forage. It is possible that some compounds with a minor content and strong activity have not been isolated and identified from the crude extract. It is also possible that the isolated phenolic compounds might create a synergistic effect that increases the bioactivity. Some compounds may contribute more to the bioactivity. Other issues include the evaluation of the antimicrobial and antioxidant activities of the phenolic compounds in biological tests and their toxicity as well as the establishment of the processes and conditions for extraction and preparation of these compounds in a large scale that need to be further studied.
